# Supplementary material for: Neural network control of focal position during time-lapse microscopy of cells
Source: Sci Rep. 2018 May 9;8:7313. doi: 10.1038/s41598-018-25458-w (PMC5943362; doi:10.1038/s41598-018-25458-w)
Supplement: Supplementary file 1 — Supplementary Information [file 41598_2018_25458_MOESM1_ESM.pdf]

Supplementary Material for

"Neural network control of focal position during time-lapse microscopy of cells"

Ling Wei[1], Elijah Roberts[1†]

March 13, 2018

[1] Department of Biophysics, Johns Hopkins University, Baltimore, MD 21218, USA

[†] Correspondence to:

Elijah Roberts

Department of Biophysics, Johns Hopkins University

Jenkins Hall 110

3400 N Charles St

Baltimore, MD 21218

Ph: 410-516-2384

Email: eroberts@jhu.edu

Supplementary Text

S1 Image-based methods to identify the image best in focus

We have tried the following three objective methods to determine the image best in focus, all of which are based on image processing. By calculating gradients, counting high-frequency components, or passing through gradient filters, the sharpness of images can be evaluated, or the edge information can be extracted. The image quality is quantified by these methods, and the one with the best quality within individual z-stacks is chosen as the image best in focus.

- Figure of merit (FOM) [1]

  A quantitative, image-based FOM is calculated for images in a z-stack, and the image with the optimal FOM is chosen as the image in focus. We used Brenner gradient as the FOM, which is shown to be more sensitive than image contrast, variance or entropy functions.

$$B = \sum_{i=1}^{N} \sum_{j=1}^{M} \big[s(i,j) - s(i+m,j)\big]^2 \tag{S1}$$

- No-reference Image Quality assessment (NR-IQA) [2]

  An image quality score is calculated in frequency domain for images in a z-stack, and the one with the highest score is the best in-focus image. Specifically, images are transformed into k-space by Fourier transformation, and the number of high frequency components (above certain threshold) is calculated. The higher the number of high frequency components is, the sharper and therefore closer to focal plane the image is.

- Edge detector (filter) [3]

  Images in a z-stack are passed through Laplacian filter, which gives the deviation of the images. Larger deviation means more edges are detected, indicating that the image is closer to the focal plane.

We analyzed all z-stacks in the testing dataset with the above techniques, and calculated their quality score defined by the respective methods. Due to the noisy background, presence of supported structures in cell chamber, and possibly too close between adjacent z-stack images, all the

image-based techniques are unable to precisely determine the image best in focus (Supplementary Fig. S2).

References

[1] Brenner, John F and Dew, Brock S and Horton, J Brian and King, Thomas and Neurath, Peter W and Selles, William D (1976) An automated microscope for cytologic research a preliminary evaluation. *Journal of Histochemistry & Cytochemistry* 24:100–111.

[2] De, Kanjar and Masilamani, V (2013) Image sharpness measure for blurred images in frequency domain. *Procedia Engineering* 64:149–158.

[3] Marr, David and Hildreth, Ellen (1980) Theory of edge detection. *Proc. R. Soc. Lond. B* 207:187–217.

Supplementary Figures

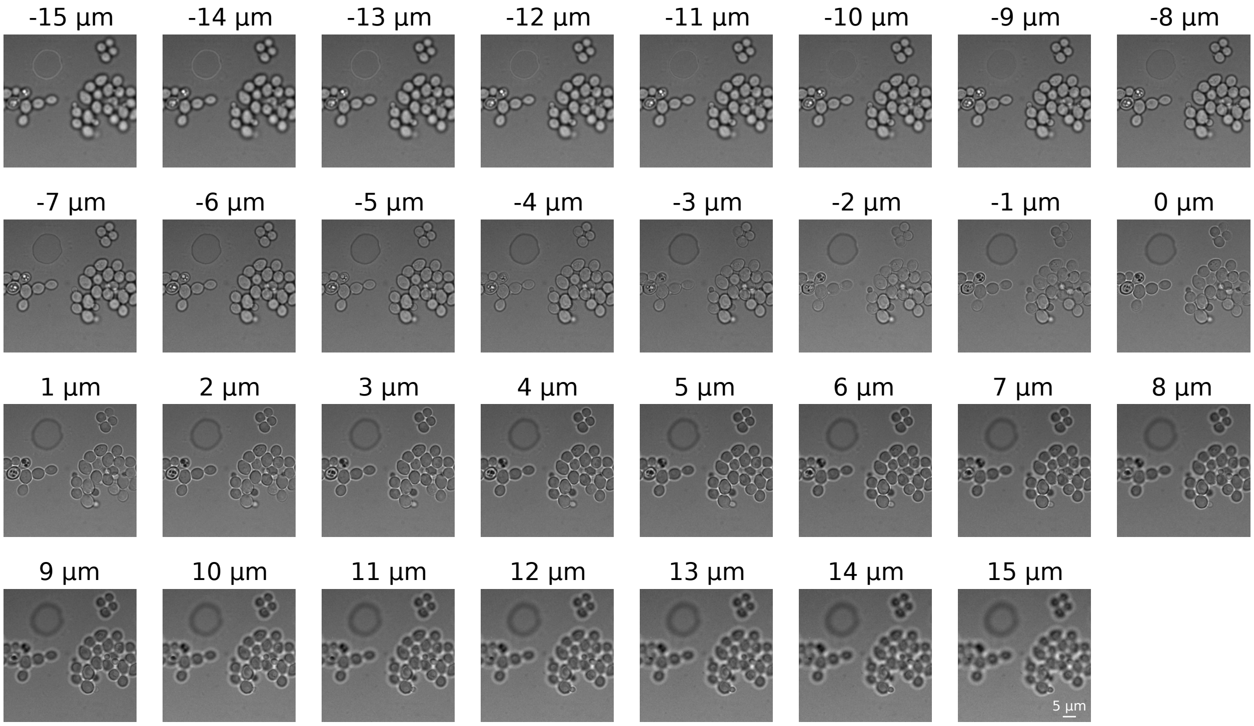

Figure S1: **Z-stack images of yeast cells.** A z-stack is composed of 31 images in steps of $\Delta z = 1\,\mu$m, including images below and above the focal plane ($z = 0$).

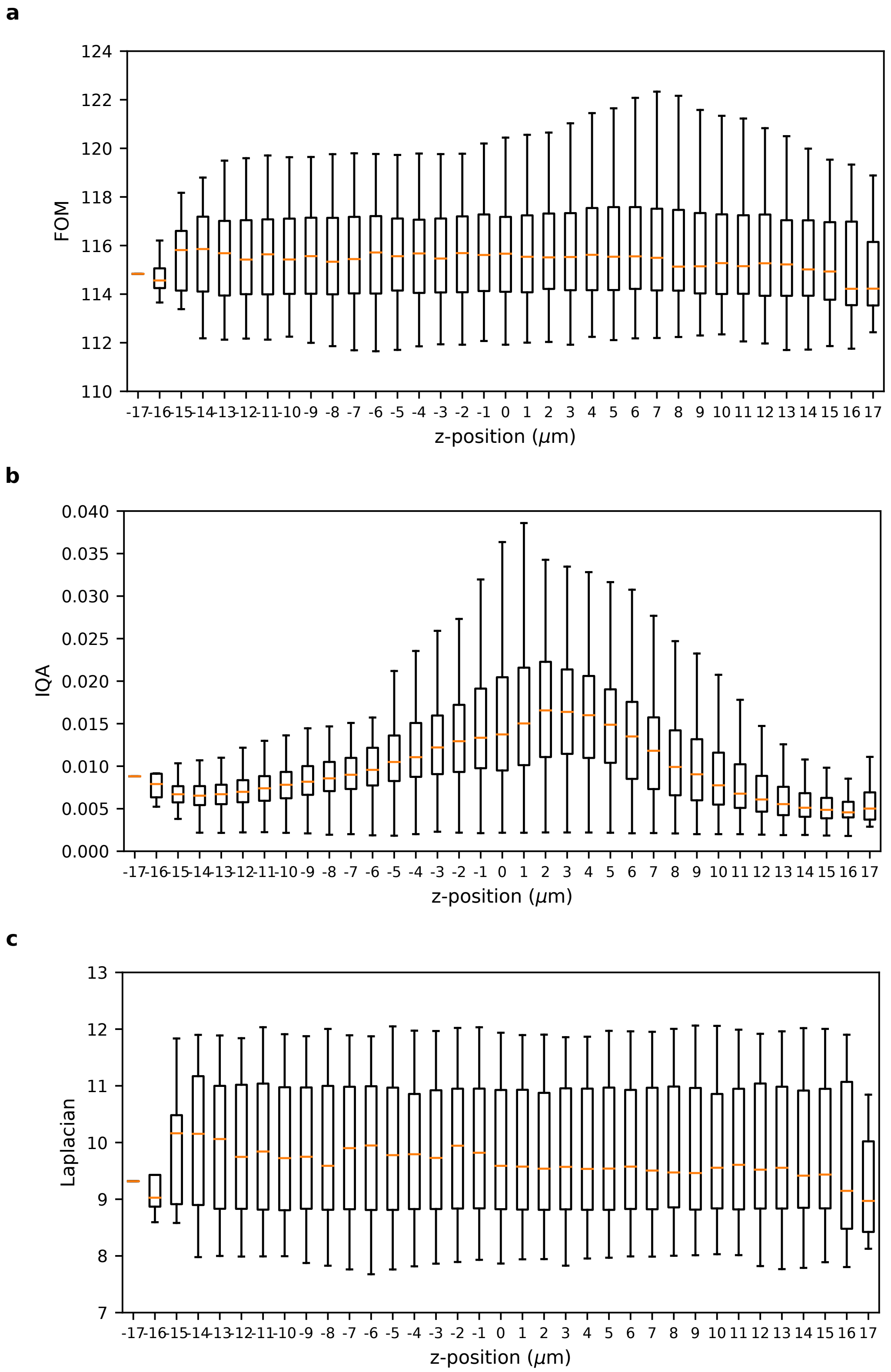

Figure S2: **Performance of non-machine learning methods in inferring image z-position.** (a) FOM–Figure of Merit, (b) IQA–Image Quality Assessment, (c) Laplacian filter.

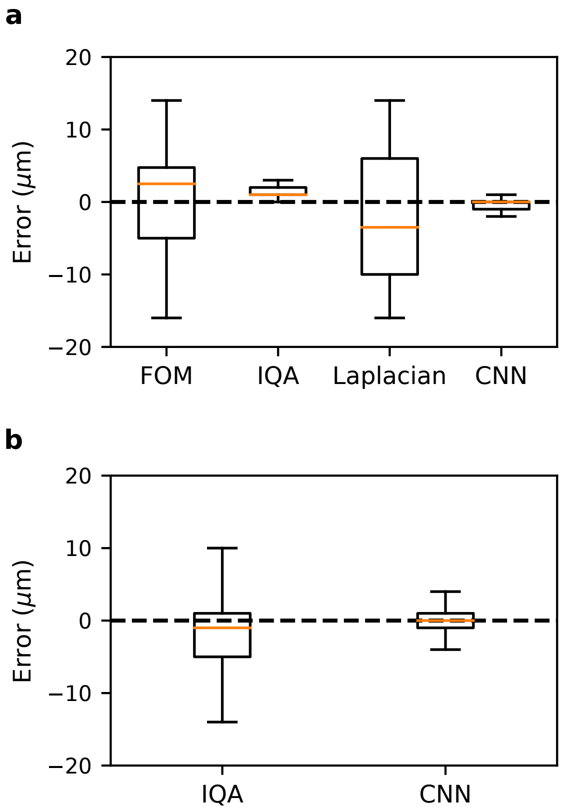

Figure S3: **Comparison of errors between non-machine learning methods and CNN.** (a) Error in determining the in-focus image given a full z-stack to analyze. (b) Error in determining the numerical z-position of an image without using any additional context.

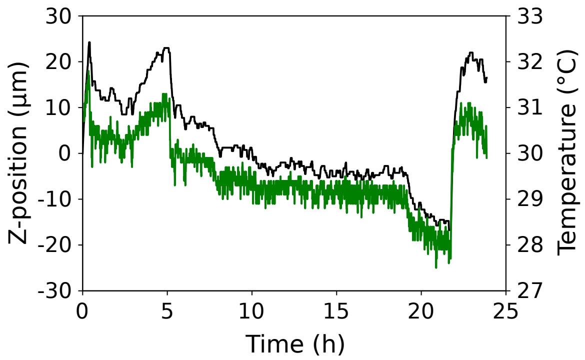

Figure S4: **Temperature-driven fluctuations of sample z-position.** Z-position of the sample (offset by its initial position, black curve) and environmental temperature (green curve) as a function of time.

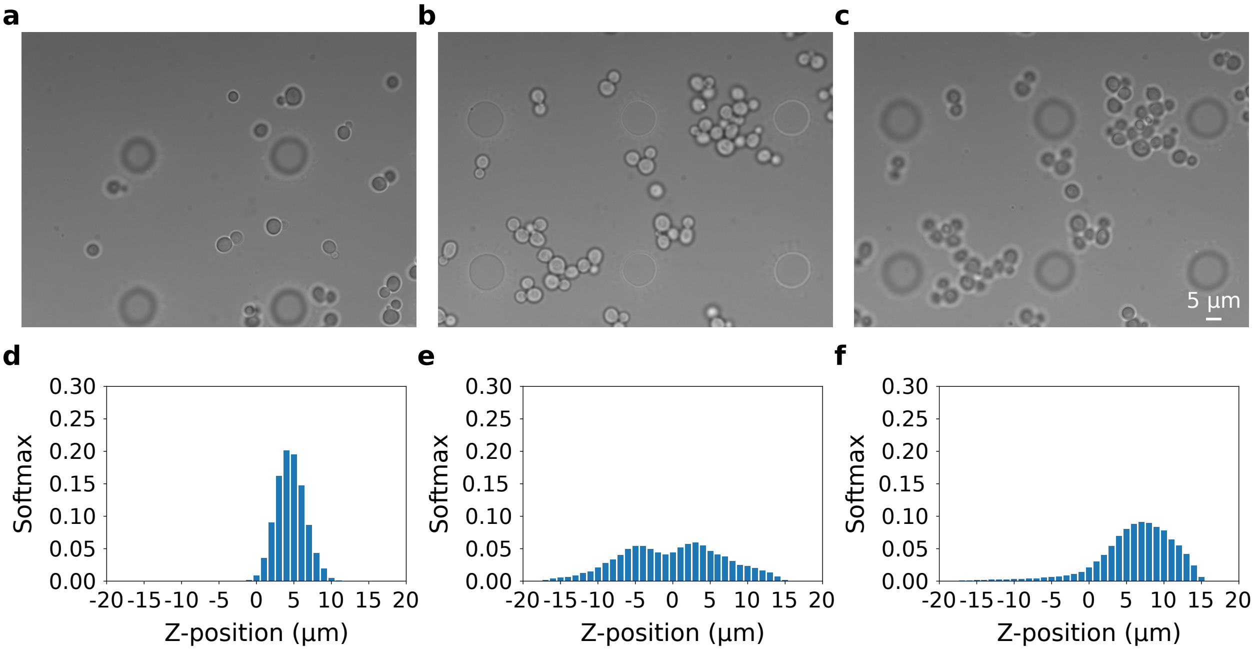

Figure S5: **Examples of failed inferences.** (a-c) Images whose real z-positions are (a) 9 μm, (b) -9 μm and (c) 12 μm. (b-f) Corresponding softmax distributions calculated by the CNN, giving the inferred z-position of (d) 4 μm, (e) 3 μm and (f) 7 μm.

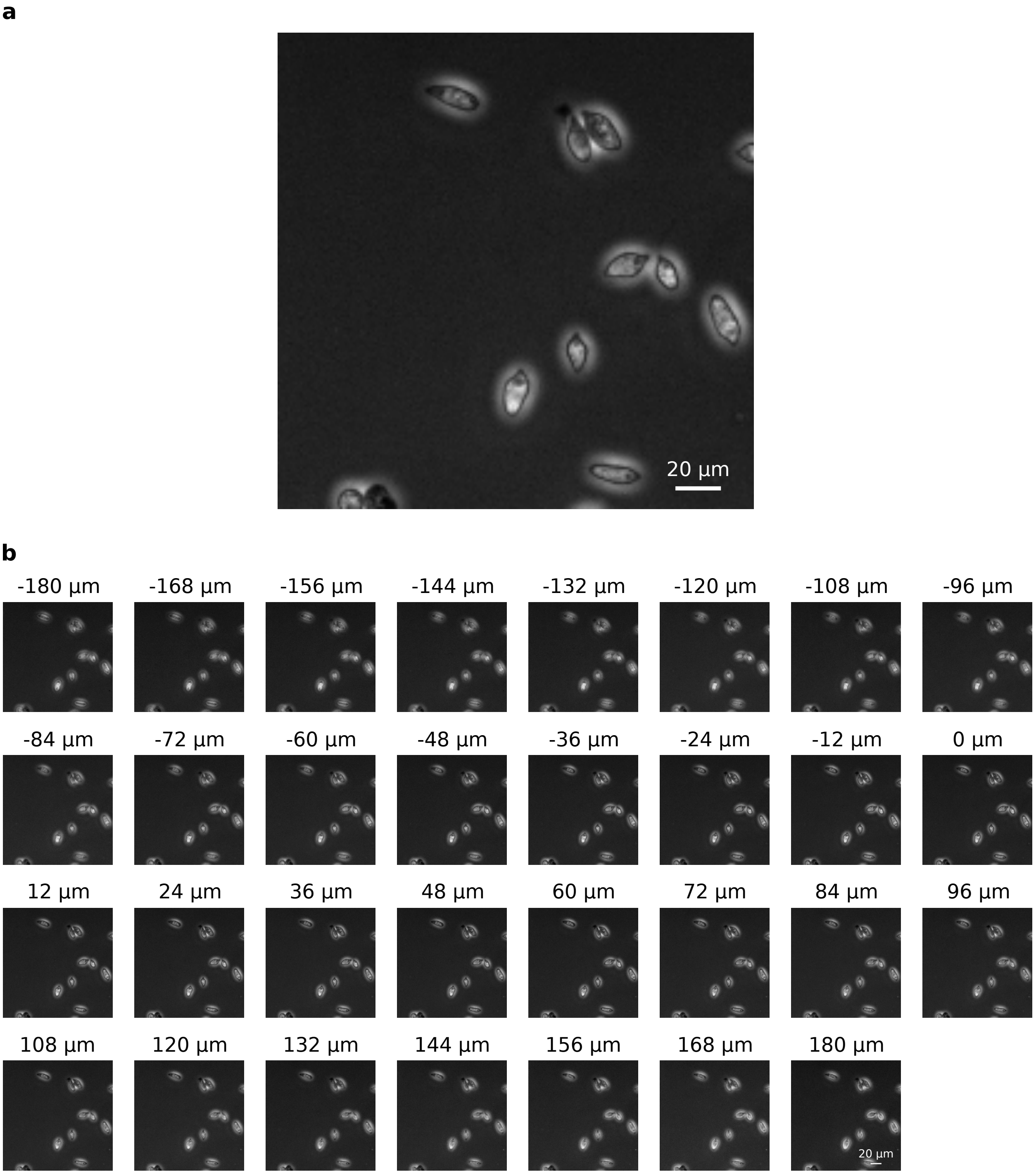

Figure S6: **Z-stack images of *Euglena* cells.** (a) An in-focus image of *Euglena* cells. (b) A z-stack is composed of 31 images in steps of $\Delta z = 12\,\mu$m, including images below and above the focal plane ($z = 0$).

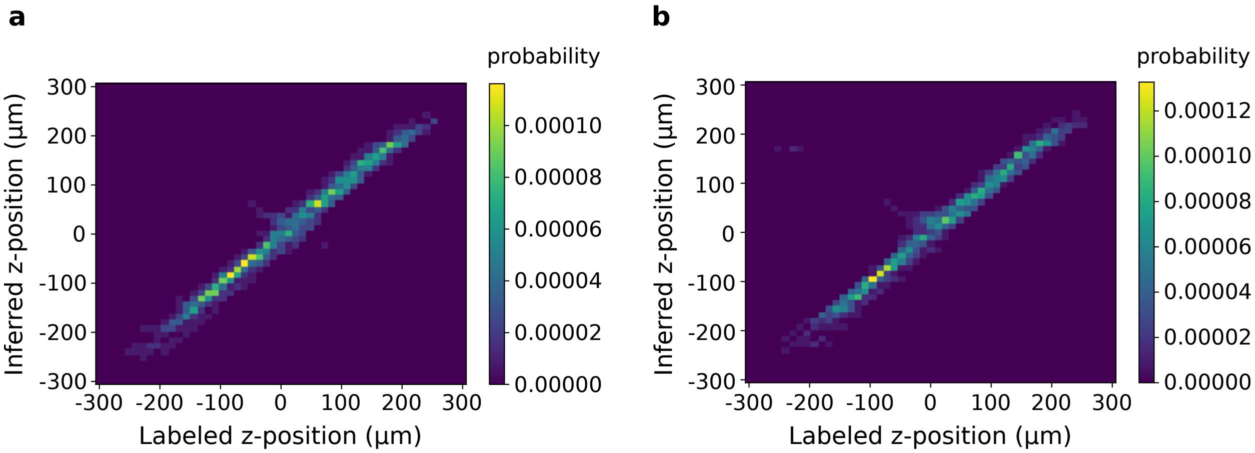

Figure S7: **Inference capbility of neural networks trained with *Euglena* cells.** The probability of inferring a z-position given the actual z-position using a CNN trained with downsampling of (a) 4X and (b) 2X. A batch size of 50 and 22 was used for 4X and 2X downsampling, respectively.

Supplementary Files

Video S8: Growth of yeast cells in microfluidics chamber. Yeast cells were grown in a microfluidic chamber for 24 hours. Real-time control of focal position was achieved by our best CNN model.

File S9: Source code for CNN training and testing. This compressed file contains python scripts that are used in training CNNs and evaluating images with trained models.
